# Persistence of Retinopathy of Prematurity in an Infant with Tetralogy of Fallot

**DOI:** 10.1155/2016/7070316

**Published:** 2016-07-20

**Authors:** Murat Gunay, Taner Yavuz, Gokhan Celik, Gunay Uludag

**Affiliations:** ^1^Department of Ophthalmology, Zeynep Kamil Maternity and Children's Diseases Training and Research Hospital, 34668 Istanbul, Turkey; ^2^Department of Pediatric Cardiology, Zeynep Kamil Maternity and Children's Diseases Training and Research Hospital, 34668 Istanbul, Turkey; ^3^Department of Ophthalmology, Koç University Hospital, 34010 Istanbul, Turkey

## Abstract

We report an infant with tetralogy of fallot (TOF) who was born at 35 weeks of gestation and of 1700 g birth weight and presented with persistent retinopathy of prematurity (ROP) at 6 months of age. Follow-up ophthalmic examinations were done at 2, 3, and 4 weeks of age. A demarcation line in Zone II was noticed on the first ocular examination done at 4 weeks of postnatal age. At 6 months of postnatal age, the infant still had an avascular peripheral retina with the demarcation line in Zone II. Even though this index subject did not have any typical risk factors for ROP, TOF seems to be the probable reason for developing as well as persistence of avascular retina.

## 1. Introduction

Tetralogy of fallot (TOF) is one of the most common cyanotic congenital heart diseases (CCHD). The prominent feature of the disease is cyanosis which depends on the disease severity [1].

It has been reported that presence of CCHD was related to the development of retinopathy of prematurity (ROP). It was indicated that cyanotic premature infants should undergo screening for ROP as other premature infants [2]. Paulus and Moshfeghi [3] demonstrated persistence of plus disease even after adequate laser therapy for ROP in a patient with TOF. Furthermore, studies have shown several retinal vascular abnormalities in association with TOF [4–6]. In the present report, we demonstrated a 6-month-old preterm infant with TOF who showed persistence of ROP with failed peripheral retinal vascularity.

## 2. Case Report

**Figure 1:**
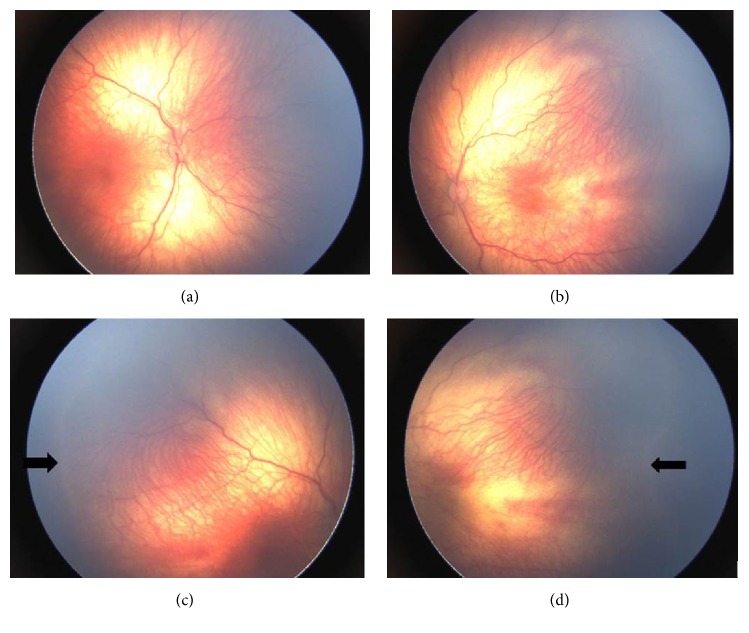
Minimal vascular tortuosity is observed in both eyes (a and b). The arrows indicate the demarcation line in Zone II along the temporal region in both eyes (c and d).

A female infant with gestational age (GA) of 35 weeks and birth weight (BW) of 1700 g was referred by a pediatrician on postnatal first month for routine examination for ROP to Ophthalmology Department of Zeynep Kamil Maternity and Children's Diseases Training and Research Hospital. The patient already was diagnosed to have TOF at Pediatric Cardiology Department at the same hospital. She also had a history of vaginal delivery after maternal induction of labor for oligohydramnios and intrauterine growth restriction (IUGR). No other significant risk factor was present before referral. At first visit, on binocular indirect ophthalmoscopic examination, a demarcation line in Zone II without plus disease was noted. ROP involved the 6 clock hours of the peripheral retina. No other abnormality was detected in anterior segment examination revealed by a hand-held slit lamp. Two weeks later, patient still demonstrated a demarcation line in Zone II. Consecutive examinations were performed with two- or three-week intervals up to a postmenstrual age (PMA) of 54 weeks in order to detect a possible progression of the retinal findings. The last examination at adjusted 6 months of age revealed persistence of the findings with demarcation line in Zone II and vascular dilatation and tortuosity (Figure 1). No additional abnormal finding was noted in both anterior and posterior segments of the eye. Fixation behavior was central and steady and was maintained in both eyes. No evidence of gaze palsy, strabismus, higher refractive error, and glaucoma was noted. Furthermore, clinical observation was still considered for TOF by the Pediatric Cardiologist due to the stable clinical course of the disease at the time of the last ophthalmologic examination.

## 3. Discussion

Tetralogy of fallot is a common form of CCHD. Although most of the patients have normal life with good function after surgical intervention, regular follow-up is crucial following surgery [7]. Several retinal abnormalities have been documented in patients with TOF. These include increased retinal vascular tortuosity, [8] retinal arterial and vein occlusions, [5, 9], and ischemic [6] and proliferative retinopathies [10].

The infant in our case had a GA of 35 weeks and BW of 1700 g. Studies from several parts of the world have reported that larger premature infants who received improper oxygen therapy can develop ROP [11, 12]. Our case has already been diagnosed with TOF at birth and required supplemental oxygen administration due to systemic hypoxia. The development of ROP might be related to this issue in our case. Supportively, Johns et al. [2] have previously reported that the presence of TOF has an association with the development of ROP. Furthermore, it has been well known that peripheral retinal avascularity is also not uncommon for ROP. But it has been shown that this situation occurs commonly in smaller premature infants with lower BW. It has been stated that spontaneous involution of ROP follows a systematic pattern wherein anteroposterior location of retinopathy changes from Zone I to Zone II or Zone II to Zone III [13]. In our case, we did not observe any sign of progression or regression pattern. Demarcation line freezed at anterior Zone II location until 6 months of age.

A reason for the development of retinal vascular abnormalities in TOF has been suggested to be ischemia. In general, oxygen saturation of the arterial blood cannot be maintained in infants with a CCHD; therefore an ischemic environment of the body arises [11]. The role of hypoxia has been demonstrated to be associated with nonperfused peripheral retina in animal models [14]. Avascular retina in our case was due to ROP. However, we can hypothesize that the infant does not have very significant risk factors for ROP and still has developed ROP. Hence, the deoxygenated blood in TOF might be responsible for altered production of factors responsible for normal retinal vascularisation and hence might have contributed to causing ROP in this case.

In another study, Paulus and Moshfeghi [3] observed persistence of plus disease in an infant with TOF after appropriate laser treatment for ROP. They indicated that vascular tortuosity following laser therapy in that case was independent of ROP regression. We also observed minimal vascular tortuosity in our case during the follow-up period.

In conclusion, we reported a larger premature infant with persistence of ROP at 6 months of age which might be associated with TOF. However, further reports are needed to better clarify the underlying mechanism of the associated retinal findings in infants with TOF.
